# Urban ants of Italy from a long-term community science-based dataset for biodiversity research

**DOI:** 10.1038/s41597-026-07235-x

**Published:** 2026-04-15

**Authors:** Matteo Bisanti, Fiorenza Augusta Spotti, Diego Gil-Tapetado, Enrico Schifani, Maria Vittoria Zucchelli, Donato Antonio Grasso, Alessandra Mori, Carlo Polidori, Cristina Castracani

**Affiliations:** 1 Department of Chemistry, Life Sciences & Environmental Sustainability (SCVSA), University of Parma, Parco Area delle Scienze, 11/a, 43124 Parma, Italy; 2 Department of Environmental Science and Policy (ESP), University of Milan, via Celoria 26, 20133 Milan, Italy; 3 Department of Ecology and Hydrology, University of Murcia, Campus Espinardo, 30100 Murcia, Spain; 4 Institute of Evolutionary Biology (CSIC – University Pompeu Fabra), Passeig Maritim de la Barceloneta, 37-49, 08003 Barcelona, Spain; 5 Museo delle Scienze di Trento (MUSE), Corso del Lavoro e della Scienza 3, 38122 Trento, Italy

## Abstract

Urban environments are increasingly recognized as important settings for biodiversity monitoring, yet empirical knowledge of insect fauna in cities remains limited. Ants are key components of urban ecosystems and useful model organisms to study the ecological effects of urbanization. Urban ant assemblages include invasive alien species, climate-sensitive taxa, and species associated with both ecosystem functioning and human environments, making them relevant to questions of biodiversity loss, sustainable urban development, and human well-being. Here we describe a nationwide dataset on urban ants in Italy, compiled through the community science project School of Ants. The dataset, accessible through GBIF, contains 4,698 occurrence records collected between 2011 and 2024 using a standardized baiting protocol (cookie-crumb–filled tubes left open for one hour). It documents 66 species and four species complexes, belonging to 21 genera and three subfamilies of the family Formicidae, and represents approximately one-fourth of the known Italian ant fauna. Data were generated through the participation of more than 6,000 volunteers and subsequently validated by taxonomic experts. Records were harmonized according to Darwin Core standards to facilitate integration with global biodiversity infrastructures. This dataset provides a spatially and temporally structured resource to investigate the effects of urbanization on ant assemblages, track dominant and non-native species. Although coverage is concentrated in Northern Italy, the standardized and accessible protocol provides a scalable framework that can be readily adopted to generate comparable urban ant datasets in other geographic contexts.

## Background & Summary

Urban areas are highly anthropogenic environments where human activities interact with a wide array of animal and plant species. Cities can therefore be regarded as complex ecosystems, shaped by unique pressures and transformations that influence community composition^1^. Urban biodiversity supports key ecosystem functions and services in cities, from regulating microclimate and nutrient cycling to supporting human well-being and opportunities for contact with nature. It therefore represents an important conservation asset, and only through systematic study and long-term monitoring can effective management and conservation actions be planned and implemented in urban landscapes^2,3^. To this aim, ants (Hymenoptera, Formicidae) represent a valuable study model for many reasons. With more than 14,000 described species worldwide^4^, ants are among the most diverse, abundant and ecologically relevant insect taxa worldwide^5–7^. They provide key ecosystem services such as soil turnover, seed dispersal, and bioindication^8–10^. Moreover, ant systematics is well resolved^11^, and their sampling is generally easy and low-cost^12^. Despite their ubiquity and ecological importance, empirical knowledge on urban ant diversity and distribution in Europe remains limited^13–18^. Documenting these patterns is crucial to assess the effects of urbanization on biodiversity, particularly in relation to climate change, habitat fragmentation, and biological invasions.

The dataset presented here provides the first large-scale description of the Italian urban myrmecofauna, with records of both species composition and distribution across multiple cities. It is designed to support comparative studies of urban ant assemblages across cities, regions and years, as well as providing a baseline for long-term ground-dwelling ant monitoring. Beyond its ecological value, the dataset establishes a baseline for green infrastructure planning, and education through the integration of standardized sampling with broad public participation.

All data originate from the community science project “School of Ants: a scuola con le formiche” (literally “School of Ants: learning with the ants”; SoA) launched in 2011 by the Insect Ethology, Ecology and Sociobiology Lab (University of Parma)^16,19^. SoA is a project in which citizen scientists conducted field data collection. No prior scientific background is required by community scientists, and the project is open to all interested participants. To ensure broad participation, different recruitment strategies are employed, such as communication campaigns, outreach events, and the development of spin-off projects. In addition, in collaboration with MUSE – Science Museum of Trento, a tailored programme has been running since 2016, involving schools at all educational levels (e.g., 6–19 years, from primary to high school), offering free teacher training and providing a curriculum to implement the project during the school year.

Sampling protocols are intentionally designed to be standardized yet accessible, employing a uniform collection kit derived from the international School of Ants framework^19^, thus ensuring comparability across sites and replicability in other regions. For each site, core metadata (GPS coordinates, sampling date, species identification, number of individuals) were recorded and harmonized according to Darwin Core standards. Taxonomic validation was performed by the researchers, and spatial and temporal consistency checks were applied. Incomplete records were verified and, when possible, enriched with additional information.

The dataset currently includes 4,698 occurrence records, collected between 2011 and 2024 across 16 of the 20 Italian regions, representing 66 ant species and four species complexes belonging to 21 genera and three subfamilies of the family Formicidae. The current checklist of the Italian ant fauna comprises 267 species and subspecies belonging to 42 genera and 7 subfamilies^20^; thus the dataset covers approximately one-fourth of the Italian ant fauna. Geographic coverage is biased towards Northern Italy, with hotspots in Trentino-Alto Adige and the Po Valley, but contributions span urban areas across most of the country.

## Methods

### Sampling by community scientists

Data were collected by more than 6,000 community scientists participating in the SoA project, using a protocol designed to be simple, standardized, and replicable across diverse contexts^16,19^.

Specifically, each sampling kit contained eight baited tubes (four with green caps and four with yellow caps, each filled with 4 mL of cookie crumbs) and a data sheet for recording site data. Green-capped tubes were placed near vegetation (e.g., grass, bushes, or trees), while yellow-capped tubes were positioned in non-vegetated spots (e.g., sidewalks, paved paths, or walls). All tubes belonging to a single kit were placed within a radius of approximately 20 steps and at a minimum distance of one metre from each other. This configuration was designed to sample distinct microhabitats (vegetated vs. non-vegetated) within the same urban site while minimizing direct interaction among baits. Tubes were left open for one hour, during which participants filled out the data sheet; afterwards, the tubes were sealed and returned with the completed sheet to the researchers.

**Fig. 1:**
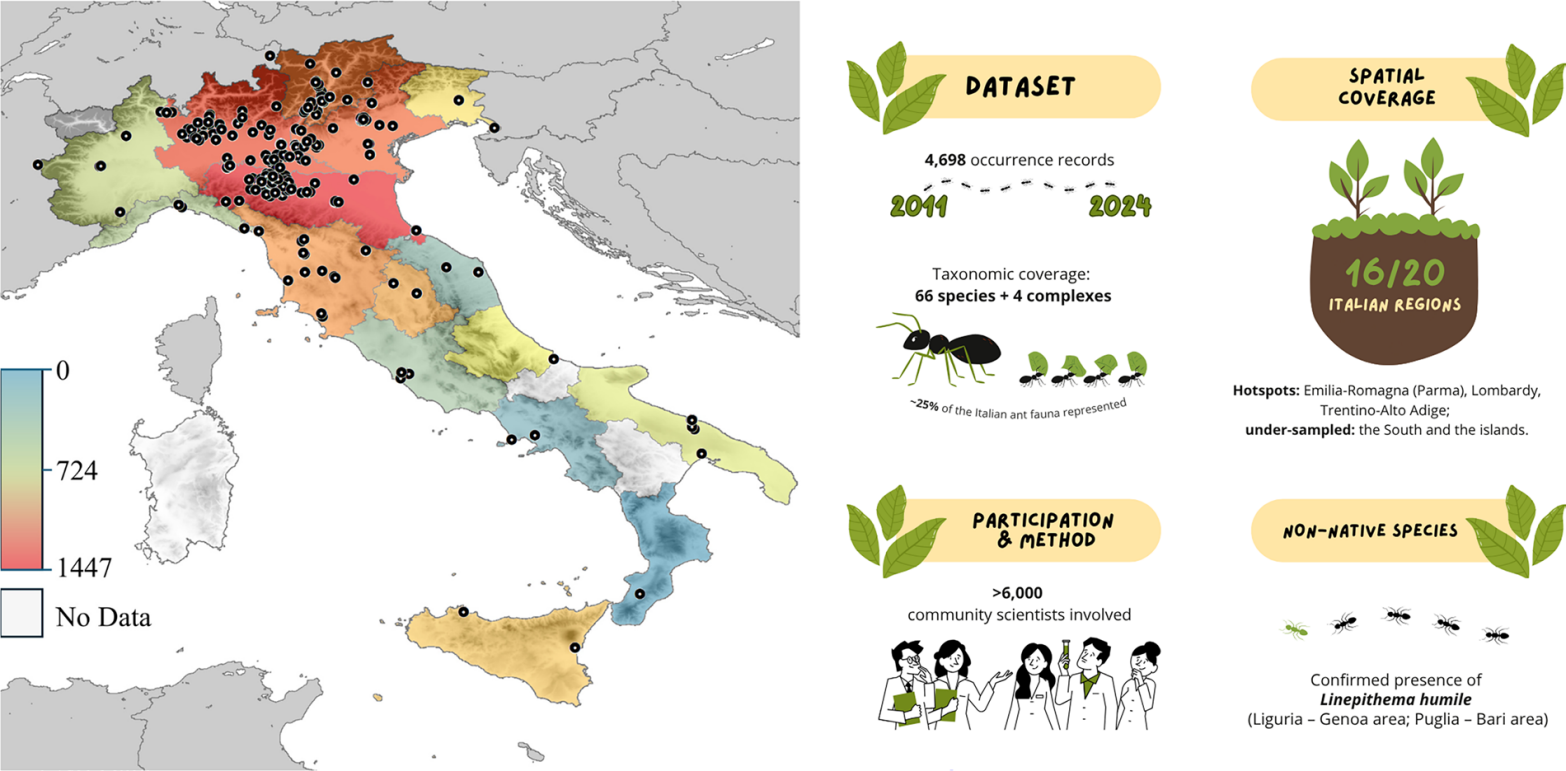
*School of Ants*—Italy dataset. Map shows sampling locations and regional record density. Summary: 4,698 records (2011–2024), 66 species + 4 complexes (~25% of the Italian fauna) from 16/20 regions, collected by >6,000 community scientists with a standardized baiting protocol; the non-native Argentine ant *Linepithema humile* is confirmed in Liguria and Apulia.

Sampling sites were chosen within urban or human-impacted areas and include both vegetated and non-vegetated zones. Private gardens were the most frequent choice for individual participants, while schools typically sampled their courtyards; public parks were also surveyed, either independently or during outreach events. At each site, one or more sampling kits were used. A “site” is defined as a discrete urban area (e.g., a single garden, park, or school courtyard) in which sampling was conducted. A “sampling event” corresponds to the simultaneous deployment of one or more kits within the same site. During a single sampling event, up to five kits were used. Sampling could be repeated over time within the same site, resulting in multiple events associated with a single geographic location. The points shown in Fig. 1 represent the geographic position of these sites. Given the bait-based nature of the protocol, the dataset primarily captures ground-dwelling ant species. As such, it is particularly suited for comparative analyses of urban ant assemblages, dominant taxa, and the detection and tracking of non-native or invasive species across space and time.

### Sample processing and data curation

The processing and identification of the samples, as well as the digitization of the corresponding data, were carried out by the researchers.

Samples were examined under a ZEISS Stemi 508 stereomicroscope (5–200 × magnification) with an Axiocam Erc 5 s and ZEISS ZEN core software. Morphometric measurements were performed when necessary to support species identification, particularly for morphologically similar taxa (e.g., within the genus *Myrmica*). These measurements were used exclusively for taxonomic validation and are not included in the published dataset. Specimens were identified to species level using specific keys^21–23^, and the number of individuals per species was recorded. Due to the difficulty of discriminating among cryptic species, in four cases identification was limited to the species complex level, namely for specimens belonging to the following cryptic species complexes: *Messor structor*, *Solenopsis fugax*, *Tapinoma nigerrimum*, and *Tetramorium caespitum*. For taxa that underwent significant taxonomic revision during the study period, records were conservatively retained at the species-complex level to ensure temporal consistency and avoid retrospective overinterpretation. All specimens are preserved for long-term reference at the University of Parma, housed within the facilities assigned to the research group and curated by the research team. Most specimens are stored in 70% ethanol, while a small subset (<10%) has been pinned and dry-preserved in entomological boxes for educational purposes. Each sample is associated with a unique kit and vial identifier, allowing traceability to collection date and geographic location as recorded in the dataset. This system ensures long-term taxonomic verifiability, particularly for species complexes and non-native taxa.

For each sample, the corresponding digital record includes taxonomic identification, individual counts per species, collection date, geographic coordinates, and the associated kit and vial codes linking the specimen to its metadata. Records were compiled annually into a unified dataset and harmonized following Darwin Core standards (https://dwc.tdwg.org/)^24^, thereby ensuring consistency across years and facilitating deposition in international biodiversity repositories such as GBIF.

### Ethics statement

Participation in the SoA project involved volunteers in the collection of non-human environmental samples (ants). According to applicable Italian legislation and research ethics regulations, activities of this kind do not fall under the definition of human-subject research and therefore do not require formal ethics committee review. Limited personal information (e.g., name and email address) was collected solely for organizational and communication purposes. These personal data were not used for research analyses, were handled in accordance with applicable data protection regulations, and are not included in the published dataset. The dataset deposited in GBIF contains exclusively environmental and taxonomic information and does not include any personal identifiers or information that could be used to identify individual participants. For activities involving minors, participation took place within regular school programs authorized by school authorities, and parents were informed about the project in accordance with standard educational procedures.

## Data Records

The dataset is available through GBIF^25^ and data are stored in Darwin Core format to facilitate integration with global biodiversity infrastructures.

In details, each record includes a unique sample identifier (“occurrenceID”), the sample’s source vial code (“catalogNumber”), the date of collection (“eventDate”), the geographic coordinates (“decimalLatitude”; “decimalLongitude”; “geodeticDatum”), the validated species name (“scientificName”), and the number of individuals observed (“individualCount”), thereby providing both occurrence and abundance information. Since all samples come from Italy, the country of origin (“countryCode”) is always indicated as “IT” for each record.

## Data Overview

The *School of Ants* – Italy dataset (Fig. 1) spans the period 2011–2024, comprising 4,698 occurrence records collected across 299 distinct urban sites in 16 of the 20 Italian regions. Here, an occurrence corresponds to a single species record within a specific tube associated with a given site and sampling event. Consequently, the same species may be represented by multiple occurrence records within a site if collected in more than one tube. According to GBIF metrics, temporal coverage shows a heterogeneous pattern, with variable sampling effort linked to the number of participating schools and local initiatives.

**Fig. 2:**
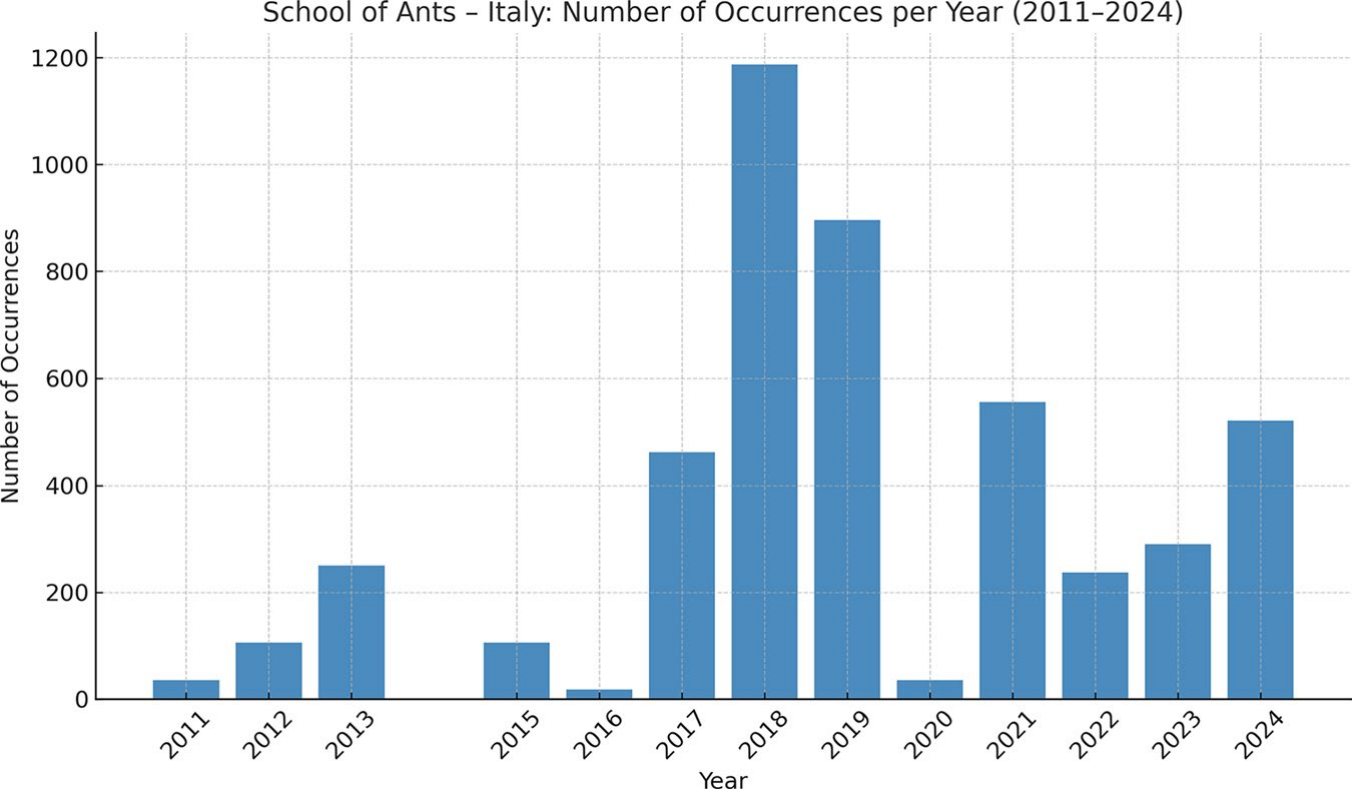
Number of occurrence records collected each year through the *School of Ants* project. Sampling effort varied substantially over time, with the highest number of records in 2018, corresponding to the *Bioblitz Lombardia* campaign, and a marked reduction in 2020 due to COVID-19 restrictions. Temporal heterogeneity largely reflects fluctuations in school participation and regional initiatives across years.

The earliest records date to 2011 (35 occurrences), with an initial rise in 2012 (106) and a marked increase in 2013 (250). Data availability remains irregular through subsequent years, with a major peak in 2018 associated with the *Bioblitz Lombardia* campaign and an almost complete absence of sampling in 2020 due to COVID-19 restrictions (Fig. 2). No records are available for 2014. This gap reflects a transitional phase in the development of the project, during which activities shifted from a predominantly local pilot initiative based in Parma and surrounding areas to a nationwide programme. In this period, the collaboration with MUSE was established and the structured school programme was developed, laying the foundation for the subsequent expansion of sampling across most Italian regions.

Spatially, records extend throughout the Italian peninsula, from Trentino-Alto Adige in the north to Sicily in the south, and from Liguria and Piedmont in the west to Friuli-Venezia Giulia and Apulia in the east. Sampling intensity is highest in Emilia-Romagna, particularly around Parma, where the project originated and remains most active. Additional hotspots include Lombardy, strengthened by regional citizen-science programmes, and Trentino-Alto Adige where sampling effort was comparatively high due to the active involvement of MUSE that has played a key role in participant recruitment and school engagement within the region. The southern regions and islands are comparatively underrepresented, highlighting priority areas for future targeted surveys.

Among the 66 recorded ant species and four species complexes, five taxa dominate the dataset: *Lasius emarginatus* (Olivier, 1792) (328 records), *L. niger* (Linnaeus, 1758) (320 records), *Formica cunicularia* Latreille, 1798 (318 records), *Crematogaster scutellaris* (Olivier, 1792) (289 records), and the *Tetramorium caespitum* species complex (1490), which is widely represented across urban and peri-urban environments in Italy. These species are among the most common and widespread in urban and peri-urban habitats across Italy. The dataset also documents the presence of the alien Argentine ant, *Linepithema humile* (Mayr, 1868), with confirmed occurrences near Genoa (Liguria) and Bari (Apulia).

Overall, the dataset contributes baseline information on spatial and temporal patterns of urban ant diversity in Italy, while remaining subject to the uneven sampling effort and associated biases typical of large-scale citizen-science initiatives.

## Technical Validation

Data quality was assured through multiple steps at both the collection and post-processing stages. Standardized sampling kits and detailed written instructions minimized variability among participants, while teacher training sessions further supported protocol compliance in schools. Only samples collected in strict accordance with the protocol — including the use of standardized kits, a sampling duration of 1 hour, and the availability of complete spatial and temporal metadata — were included in the final dataset. Records not meeting these criteria were excluded.

Taxonomic validation was carried out exclusively by the research team. During data harmonization, species names were revised and updated to reflect current taxonomy, accounting for nomenclatural changes that occurred during the data collection period. Particular attention was given to cryptic or taxonomically challenging groups. In cases where reliable species-level identification was not possible (e.g., *Messor structor*, *Solenopsis fugax*, *Tapinoma nigerrimum*, and *Tetramorium caespitum*), records were conservatively assigned to species complexes to ensure taxonomic consistency and avoid overinterpretation.

Consistency checks were applied to all metadata fields to validate data structure and internal coherence. Spatial validation involved cross-checking GPS coordinates against administrative boundaries to identify and correct erroneous records. Temporal validation ensured that collection dates were compatible with project timelines and school calendars. Completeness checks identified missing or ambiguous information, which was verified with participants when possible or otherwise excluded.

Finally, all records were harmonized and formatted according to Darwin Core standards. Field mapping and controlled vocabularies were reviewed manually to ensure that data transformation and standardization were performed correctly, guaranteeing compatibility with GBIF requirements. Together, these procedures ensure that the dataset is taxonomically robust, spatially and temporally coherent, and suitable for comparative biodiversity analyses.

## Usage Notes

The dataset supports a broad range of applications in ecology and conservation. It can be employed for species distribution modelling of urban ants at both national and regional scales, for the early detection of non-native taxa, and for evaluating responses of urban ant assemblages to urban green infrastructure. It also provides a temporal benchmark for long-term monitoring of urban biodiversity, particularly in relation to climate change and land-use dynamics and serves as an educational resource for schools and citizen science initiatives.

However, as with many citizen science datasets, some limitations should be considered. Geographic coverage is biased towards Northern Italy, reflecting volunteer availability, and temporal coverage is uneven, with greater sampling effort in years of higher school participation. Moreover, the exclusive reliance on baits underrepresents arboreal or hypogeic species. These limitations are documented in the metadata and should be considered when integrating the dataset with other sources when designing comparative analyses. Presence data in this dataset are robust and taxonomically validated, providing a reliable basis for ecological and biogeographic analyses. However, absence data are constrained by heterogeneous sampling intensity across space and time, and their interpretation should take into account potential gaps. Future targeted surveys could complement the dataset and refine absence-based assessments. In addition to occurrence data, the dataset includes abundance information (“individualCount”), representing the number of individuals of each species collected per tube. While these values do not constitute standardized population density estimates, they provide a proxy of recruitment intensity to a controlled food resource. Given the social nature of ants and their recruitment behaviour, these data can support comparative analyses of foraging dynamics, dominance patterns, and the relative competitive performance of native and non-native species across urban contexts.

At present, the GBIF dataset does not include standardized habitat or microhabitat descriptors for each sampling event, which limits its direct use for fine-scale habitat comparisons. Future releases of the School of Ants – Italy dataset will aim to incorporate site- and habitat-level information wherever these data are available from ongoing and future sampling campaigns.

## Data Availability

All data described in this Data Descriptor are available through the Global Biodiversity Information Facility (GBIF) repository as the dataset “Myrmecological Collection from Italian Citizen Science Project ‘*School of Ants*’^25^ at https://www.gbif.org/dataset/92c064da-7d2e-4079-b491-f48c54eacb63. Users are encouraged to cite this dataset using the data citation provided in the References section.

## Code availability

No custom code was used to generate or process the dataset described in this Data Descriptor. All data handling steps are described in the Methods and Technical Validation sections.
